# Theranostic Pretargeting Drug Delivery and Imaging Platforms in Cancer Precision Medicine

**DOI:** 10.3389/fonc.2020.01131

**Published:** 2020-07-22

**Authors:** Sudath Hapuarachchige, Dmitri Artemov

**Affiliations:** ^1^The Russell H. Morgan Department of Radiology and Radiological Science, The Johns Hopkins University School of Medicine, Baltimore, MD, United States; ^2^Department of Oncology, The Sidney Kimmel Comprehensive Cancer Center, The Johns Hopkins University School of Medicine, Baltimore, MD, United States

**Keywords:** theranostics, cancer therapy, pretargeted therapy, drug delivery, nanomedicine, bioorthogonal click chemistry

## Abstract

Theranostics are nano-size or molecular-level agents serving for both diagnosis and therapy. Structurally, they are drug delivery systems integrated with molecular or targeted imaging agents. Theranostics are becoming popular because they are targeted therapeutics and can be used with no or minimal changes for diagnostic imaging to aid in precision medicine. Thus, there is a close relation between theranostics and image-guided therapy (IGT), and theranostics are actually a subclass of IGT in which both therapeutic and imaging functionalities are attributed to a single platform. An important theranostics strategy is biological pretargeting. In pretargeted IGT, first, the target is identified by a target-specific natural or synthetic bioligand followed by a nano-scale or molecular drug delivery component, which form therapeutic clusters by *in situ* conjugation reactions. If pretargeted drug delivery platforms are labeled with multimodal imaging probes, they can be used as theranostics for both diagnostic imaging and therapy. Optical and nuclear imaging techniques have mostly been used in proof-of-concept studies with pretargeted theranostics. The concept of pretargeting in theranostics is comparatively novel and generally requires a confirmed overexpression of surface receptors on targeted cells/tissue. In addition, the receptors should have natural or synthetic bioligands to be used as pretargeting components. Therefore, applications of pretargeting theranostics are still limited to several cancer types, which overexpress cell-surface markers on the target cancer cells. In this review, recent discoveries of pretargeting theranostics in breast, ovarian, prostate, and colorectal cancers are discussed to highlight main strengths and potential limitations the strategy.

## Introduction

Theranostics is a rapidly developing field that combines the unique opportunities offered by nanotechnology with personalized medicine to provide significantly improved treatment efficacy with reduced off-target effects through the specific delivery of therapy to targeted tissues. Theranostic approaches combine imaging that uses one of the non-invasive imaging modalities, with specific delivery of therapeutic components, which can be based on different biophysical and biological principles. Theranostics can be synthesized to have optimal delivery properties, low renal clearance, reduced immunogenicity and antigenicity (for example by PEGylating the surface of theranostic nanoparticles), and high capacity for therapeutic agents, which is required given the limited concentrations of specific molecular markers expressed on cancer cells.

### Theranostics

Theranostic probes can be used for both diagnostic imaging and therapy (1). For a truly theranostic application, imaging and therapeutic molecules should be parts of a single platform functionalized with various moieties for specific recognition of molecular targets, imaging markers, and therapeutic compounds. One of the major problems in achieving efficient treatment in this strategy is the uniform delivery and distribution of theranostics with therapeutic cargo in the tumor or sufficiently strong bystander effects of therapy (such as for hyperthermia). As the distribution of nano-scale drug delivery platforms is primarily driven by the passive diffusion facilitated by the enhanced-permeability-retention (EPR) effect, which is present in some solid tumors, optimization of the molecular size, and circulation time of nanoplatforms is of paramount importance for the success of treatment. However, there are additional biological delivery barriers, such as the desmoplastic tumor microenvironment (2) and increased interstitial pressure due to dysfunctional lymphatics in solid tumors (3), which can modulate the delivery. The presence of a functional blood-brain barrier in brain tumors presents an entirely new set of problems with delivery, which is beyond the scope of the current review. Imaging can help to evaluate the uniformity of delivery, but, to achieve high efficacy, the design of a therapeutic system must ensure the effective delivery and distribution of theranostics to all malignant cells within the cancer.

While the initial distribution of theranostics in tumors is driven by passive processes and needs to be optimized based on the intrinsic physicochemical properties of platform, specific retention and cell delivery is typically controlled by active targeting of theranostic to specific molecular targets present on cancer cells using high-affinity molecules, such as antibodies, antibody fragments, affibodies, peptides, etc. It is important to note that, while unique, highly specific molecular targets are preferable for the application of highly cytotoxic theranostics, the initial delivery of theranostics rely on the tumor-specific EPR effect. The “binding site barrier” is a critical factor in targeted drug delivery (4, 5). This issue can be circumvented in theranostic design using biomolecules as platforms and keeping the size of components in subnano level (6).

### Pretargeting Theranostic Approach

Important criteria that should be fulfilled for a truly theranostic platform is that the imaging data must provide meaningful information, which can be used to make critical decisions regarding the therapeutic procedure. In pretargeting therapy, the first component is typically used for labeling the target and obtaining the location and size (perhaps tumor stage) of the tumor. Since the pretargeting component is not cytotoxic, its long circulation (and possible weak non-specific binding) does not generate side effects, while providing improved distribution in the tumor. After the target is confirmed, the second therapeutic components can be administered chemoselectively cross-linking the first pretargeting component on cell surface.

**Figure 1:**
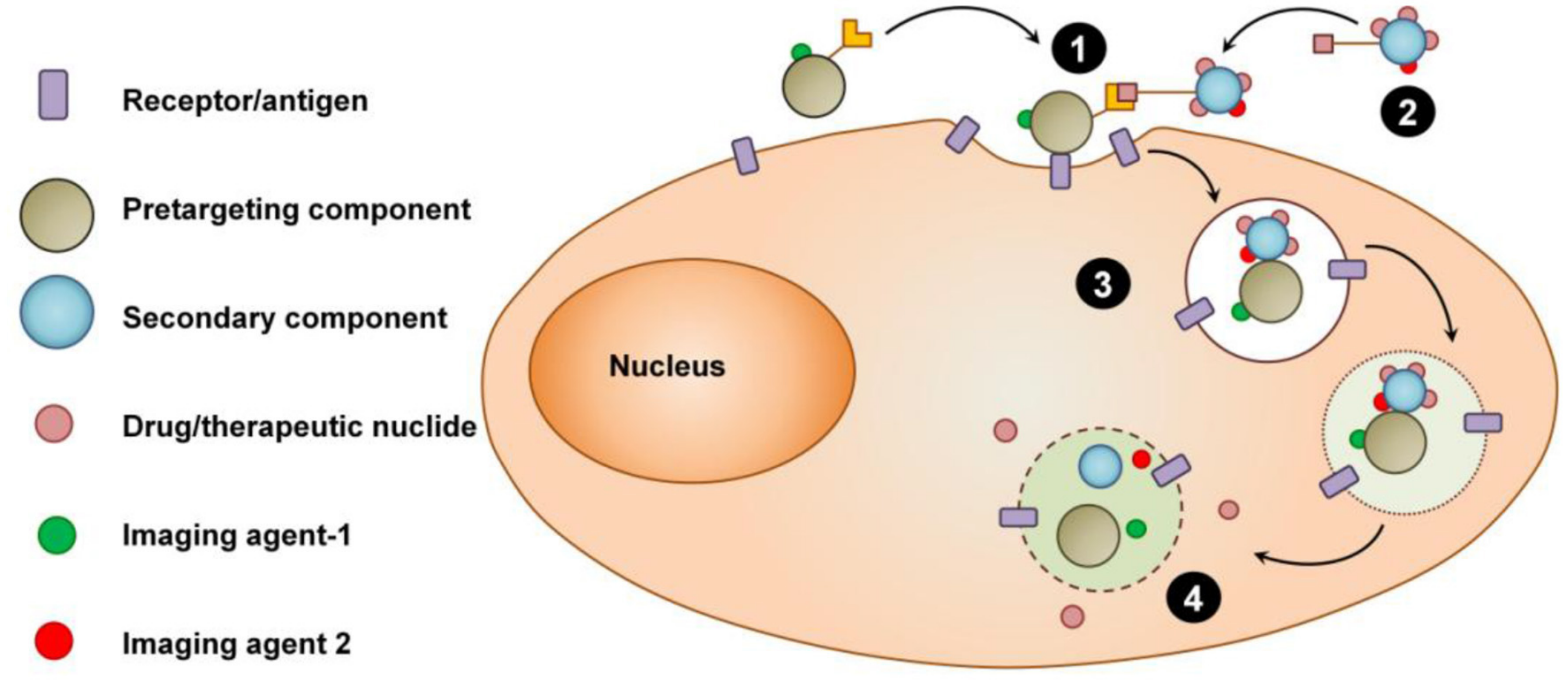
Schematic view of the concept of pretargeting theranostic strategy. (1) Image-guided pretargeting. (2) Therapeutic/radionuclide delivery step. (3) Internalization of the complex of pretargeting-receptor-delivery components. (4) Intracellular release of therapeutics or radionuclides.

Contemporary targeted drug delivery vehicle such as ADCs, with an imaging marker may provide imaging information about the delivery and distribution of the platform in the tumor. Micro-dosing can also be used to track the delivery and image the distribution prior to the administration of the therapeutic dose (Figure 1). However, while these approaches can be considered theranostic, in the first case, imaging would only provide secondary information that cannot be used to change and/or optimize the treatment protocol, and, in the latter case, imaging is limited to a significantly reduced dose and the therapeutic dose of nanomedicine is delivered without image guidance.

### *In situ* Conjugation Methods in Pretargeting Theranostics

The conjugation between pretargeting component and the therapeutic delivery component occurs in the biological system in physiological conditions. This *in situ* conjugation method should be fast and proceed at 37°C without releasing toxic byproducts. Avidin-biotin interaction is one of the early stage *in situ* conjugation techniques used for pretargeted imaging and therapy (7). Avidin is a tetrameric protein which binds biotin with high affinity. Since avidin is immunogenic and has a broad non-specific binding, this conjugation method can lead to adverse biological effects and toxicities. Bioorthogonal click chemistry is an alternative conjugation method widely used nowadays for *in situ* conjugation. *Trans*-cyclooctene/tetrazine bioorthogonal click reaction is extremely fast and widely used for pretargeting conjugation compared to other reactions in this category such as, copper-free azide-cyclooctyne click chemistry and Staudinger ligation (8, 9). Specific interaction between peptide nucleic acids, which are non-natural DNA/RNA analogs, can also be used for pretargeting approach (10). These compounds are metabolically stable and show low non-specific binding in healthy tissues.

### Imaging Strategy in Theranostic Systems

A wide variety of imaging modalities used for theranostics include optical (fluorescence or bioluminescence), nuclear (PET or SPECT), ultrasound, photoacoustic, magnetic particle, and MR imaging techniques (11, 12). The imaging results pretargeting component can be directly used to (i) verify the expression of surface receptors at the target site and (ii) precisely time the administration of the second, drug-delivery component, based on high specific accumulation of the pretargeting component at the target site and clearance from the rest of the body. Expression of receptors can be variable between the primary tumor and distant metastatic sites and not easily accessible by biopsy. Therefore, images from pretargeting component provide a positive confirmation of the receptor expression and a “go” signal for the administration of the cytotoxic drug carrier. Imaging of the drug carrier is equally important, as it can provide positive confirmation of the successful delivery and retention of the cytotoxic component in the tumor. These imaging results can also be used to correlate the delivery and treatment outcomes and to support their use as imaging markers of response. The optical imaging is widely used in preclinical drug developments because it's fast, convenient, non-invasive, safe, and cost-effective (13). High tissue absorbance and scatter are the major disadvantages in optical imaging in deep locations; however, this issue can be partly overcome in preclinical imaging using near infra-red (NIR) dyes (λ_ex_ in the range of 650–900 nm) (14). In spite of aforementioned problems, NIR optical imaging became a popular imaging technique in pretargeting studies for visualizing the target and the probe and it can provide sufficient information about the target-uptake and biodistribution of the pretargeting component, as well as the tumor dimension and location information in cancer therapy (15).

Nuclear imaging techniques are highly feasible for imaging pretargeting delivery because of excellent sensitivity, quantitative images, and the possibility of using in radiotherapy (16). Nuclear imaging provides sensitivity in the low nM to pM range, but has low spatial resolution and requires an additional anatomical imaging modality, such as CT or MRI, for anatomical reference. PET and SPECT imaging techniques are translational and can be used in both animals and patients. PET provides the highest sensitivity combined with improved spatial resolution; however, it is impossible to differentiate between two tracers with similar half-lives. SPECT provides an intrinsic ability for multi-isotope imaging based on different energy of γ emission (17). However, it is not commonly used for tracking of pretargeting components because of lower sensitivity and spatial resolution. However, it has been used for imaging the second therapeutic component of the system. For instance, in image-guided pretargeting radioimmunotherapy (PRIT), the targeted cells are labeled with antibodies conjugated with TCO groups and treated with SPECT imaging/therapeutic radionuclide conjugated with tetrazine. Pretargeting radiotherapy is one example of combining nuclear imaging to circumvent the use of long-lived radionuclides that is a necessity for sufficient tumor accumulation and target-to-background ratios using conventional approaches (16).

The imaging signals of the second component are mostly used for the biodistribution and pharmacokinetic evaluation and not for detection and evaluation of the tumor, hence the poor contrast and spatial resolution of SPECT imaging can be tolerated. MRI provides high spatial resolution and excellent soft-tissue contrast, but has a moderate sensitivity and requires concentrations of the imaging probe in high micromolar range. Fluorescence optical imaging provides outstanding sensitivity and resolution, but can only be used in superficial locations, such as for image-guided surgical resection, or in optically transparent tissues, such as ocular imaging. Examples of anticancer theranostics applications, which use these multiple imaging modalities are: PET and NIR optical imaging (18); SPECT (19), and MRI (20).

It is important to note that combining a therapeutic platform with short-lived imaging probes presents significant problems because of the short lifetime of the preparation, and consequently, with the logistics of treatment administration and monitoring even if the medication can be formulated in a kit form for rapid radiolabeling (21). Therapeutic modalities used in theranostic nano-platforms include cytotoxic drugs (22–24), radioisotopes (25), optical absorbers for photothermal or photodynamic therapy (26), and phototherapy (27). Specific enzymes can be delivered to activate prodrugs in the context of prodrug therapy (28). Magnetic NPs are therapeutic cargo for magnetic hyperthermia (29), and antibodies, adjuvants, or vaccines can be used for cancer immunotherapy (30). Most often, imaging techniques used in pretargeting theranostics rely on complementary optical and nuclear imaging modalities, such as fluorescence, bioluminescence, PET and SPECT imaging.

## Examples of Pretargeted Theranostics

### Pretargeted Theranostics in Breast Cancer

**Figure 2:**
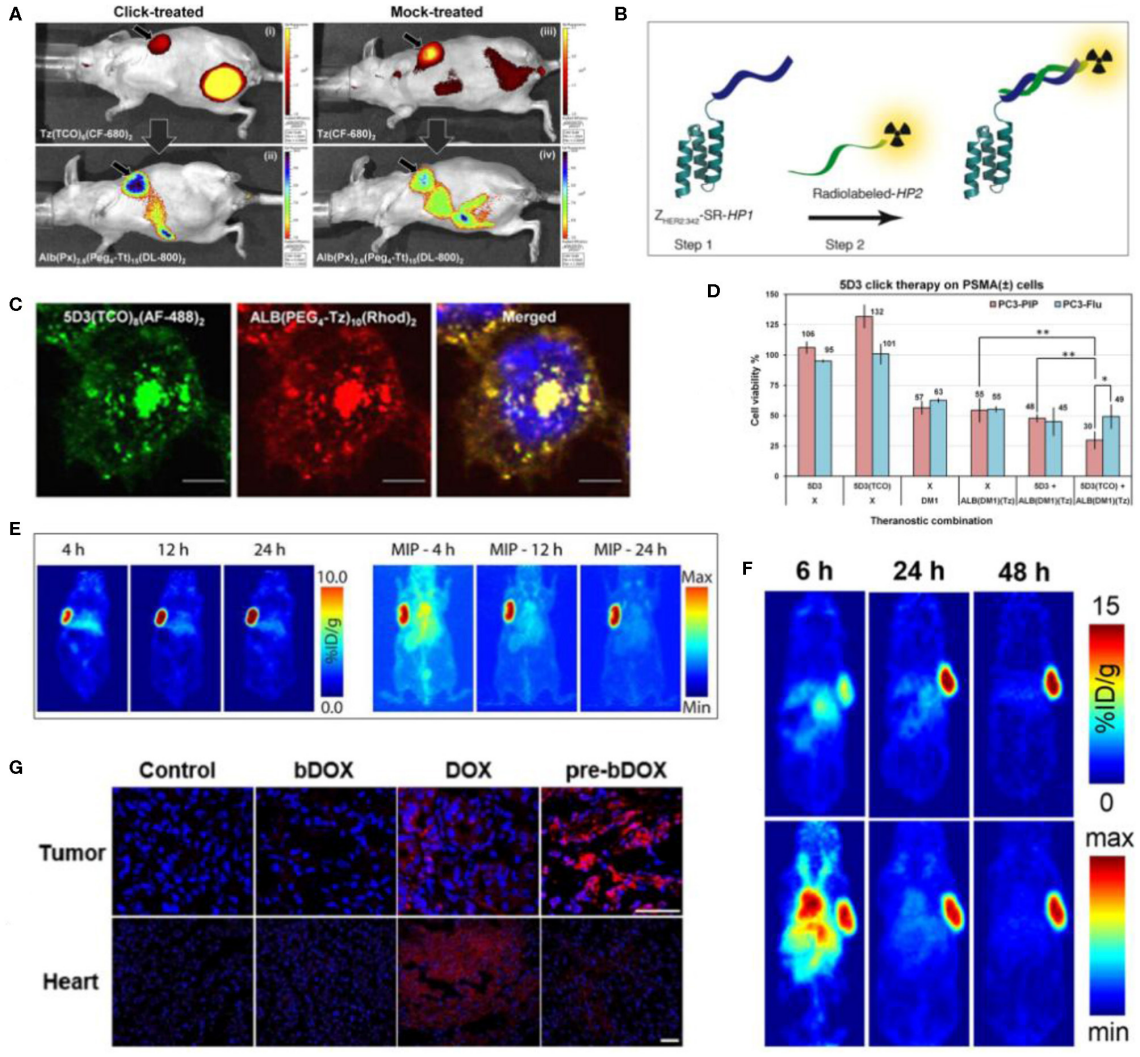
Examples of pretargeted theranostic approaches. **(A)** *In vivo* Xenogen fluorescence images after 8 h post-injection of the secondary component (after 20 h post-injection of pretargeting component). (i) Distribution of pretargeting component Tz(TCO)_6_(CF-680)_2_ and (ii) tumor uptake of delivery component Alb(Px)_2.6_(Peg_4_-Tt)_15_(DL-800)_2_. (iii) Distribution of control Tz(CF-680)_2_ and (iv) Alb(Px)_2.6_(Peg_4_-Tt)_15_(DL-800)_2_ in a mock-treated mouse (23). **(B)** Schematic view of the strategy. In step-1 Z_HER2:342_-SR-*HP1* is injected and labeled the HER2(+) tumor cells. Next, the secondary probe *HP2* is injected in step 2. The PNA sequence in *HP2* is matching with *HP1* and hybridized to the pretargeting component bound on cell surface (36). **(C)** Confocal fluorescence microscope images of pretargeted theranostic approach in PSMA(+) PC3-PIP cells. Distribution of 5D3(TCO)_8_(AF-488)_2_ (green), ALB(PEG_4_-Tz)_10_(Rhod)_2_ (red), and Hoechst 33342 nuclear counterstaining (blue) (magnification × 100, bar: 30 μm) (22). **(D)** *In vitro* therapeutic study of 5D3(TCO)_8_. The combination of 5D3(TCO)_8_ and ALB(DM1)_3.3_(PEG_4_-Tz)_10_ exhibited a selective and enhanced toxicity in PSMA(+) PC3-PIP cells compared to the combination of non-functionalized 5D3 and ALB(DM1)_3.3_(PEG_4_-Tz)_10_ or treatment with a free DM1 or ALB alone. (*p < 0.05, **p < 0.005) (22). **(E)** Pretargeting PET images of planar and maximum intensity projection (MIP), left and right, respectively in subcutaneous SW1222 tumor bearing nude mice. HuA33-Dye800-TCO was injected (100 μg; 0.66 nmol) and after 48 h, ^64^Cu-Tz-SarAr was injected. Coronal slices selected from the center of the tumors are shown (37). **(F)** PET images of the athymic nude mice with subcutaneous SW1222 tumor xenografts. The mice were first injected with huA33(TCO)_2.4_, followed after 24 h by the injection of [^64^Cu]Cu-SarAr-Tz and after 24 h by the injection of [^177^Lu]Lu-DOTA-PEG_7_-Tz. Images are shown at 6, 24, and 48 h after the injection of [^64^Cu]Cu-SarAr-Tz. Top row: Coronal planar images through center of the tumor. Bottom row: maximum intensity projections (MIP) (38). **(G)** Confonal fluorescence images of frozen sections showing doxorubicin drug uptake in tumor and heart tissues after the administration of PBS, un-pretargeting bDOX, free doxorubicin or pretargeting bDOX. Blue color represents DAPI-stained nuclei, and pseudo-red color represents fluorescence from DOX or bDOX. The scale bar: 50 μm. *p < 0.05 (pre-bDOX: pre-targeted bDOX) (39).

Breast cancer is the most prevalent malignancy in women in the United States, and ~20–30% of human BrCa overexpress HER2 receptors, a molecular marker that correlates with cancer aggressiveness, metastasis, and poor prognosis. HER2-overexpressing BrCa are treated with the humanized anti-HER2 monoclonal antibody, Trastuzumab (Tz), which is highly efficacious, but unfortunately, trastuzumab resistance develops over long-term use (31–33). Trastuzumab-based ADC, T-DM1, was developed for treatment of resistant tumors by directly conjugating the chemotherapeutic drug, mertansine, on the antibody to boost cytotoxicity (34). A potential problem with ADCs is their intrinsically highly toxicity, which can cause non-specific off-target effects in normal tissues due to their long circulation times (35). Hapuarachchige et al. (23, 24) has reported a pretargeting strategy driven by bioorthogonal click chemistry to circumvent this issue. In these studies, HER2(+) cancer cells were prelabeled with a trastuzumab mAb, functionalized with a bioorthogonal, click-reactive, TCO group, and the drug-carrier albumin component with the complementary click-reactive Tt group is delivered after free antibodies have cleared the body, to ensure that the toxic drug component accumulates only at the tumor site (Figure 2A). The strategy has been evaluated in human BrCa BT-474 cells and their HER2(+) subcutaneous tumor mouse models. Mice were administered with Tz(TCO)_6_(CF-680)_2_ image-guided pretargeting component followed by Alb(Px)_2.6_(Peg_4_-Tt)_15_(CF-750)_2_ drug delivery component. The result revealed that pretargeting approach driven by bioorthogonal click chemistry has higher therapeutic efficacy than the treatment by drug delivery component alone. This therapeutic regimen, when integrated with molecular imaging modalities, can become an effective theranostic tool that will eventually steer the development of cancer regimens toward precision, individuality, and safety.

### Pretargeted Theranostics in Ovarian Cancer

Ovarian cancer is the deadliest gynecological cancer in women; hence, the early detection and treatments are vitally important (40). Efforts have been taken to developed drugs to treat ovarian cancers overexpressing estrogen receptor (ER) and HER2. However, long term use of these novel therapies gains drug resistance. Therefore additional therapeutic approaches, such as radioimmunotherapy are needed to be developed for ovarian cancer to overcome the chemo-resistance issues (41). Affibody is a relatively low molecular weight high-affinity protein, which can be used instead of monoclonal antibodies for diagnostic imaging and therapy. Honarvar et al. (36) has developed a HER2 specific affibody conjugate and complementary secondary imaging component and evaluate in HER2(+) ovarian cancer xenografts. They have synthesized Z_HER2:342_-SR-*HP1* and used it as the pretargeting component with 15-mer *HP1* peptide nucleic acid moiety to recognize complementary secondary component, ^111^In-/^125^I-*HP2* (Figure 2B). The results revealed that the HER2(+) tumor uptake of pretargeting, Z_HER2:342_-SR-*HP1* was significantly higher than HER2 low expressing cells. In the pretargeting approach, accumulation of ^111^In-*HP2* after the administration of Z_HER2:342_-SR-*HP1* was significantly higher compared to the administration of ^111^In-*HP2* alone. In regular radioimmunotherapy, the fast clearance of the secondary component, ^111^In-*HP2*, results in low tumor uptake in bones. Pretargeting strategy enhances the accumulation and retention of the radiotherapeutic agent at the target.

### Pretargeted Theranostics in Prostate Cancer

Prostate cancer is one of the most devastating cancer types in men. Yearly more than 150,000 patients are diagnosed with prostate cancer and over 30,000 among them die. Despite existing therapies in prostate cancer management, novel therapeutic approaches and drug delivery strategies are still required to save these patients and eradicate the decease completely (42–44). Prostate-specific membrane antigen (PSMA), a type II membrane protein, is highly expressed in aggressive prostate cancer (PCa) (45–48). In our studies, we have used 5D3 mAb, a novel anti-PSMA mAb which exhibits a 10-fold higher binding affinity on PSMA compared to other known anti-PSMA mAb, such as J591 and 7E11 mAbs (49, 50). 5D3 mAb shows fast internalization after complexing with PSMA; however, this strategy has been successfully applied and proven *in vitro*.

Hapuarachchige et al. (22) has reported a promising pretargeting theranostic approach for treating PSMA-overexpressing prostate cancer using 5D3 mAb. Here, 5D3 conjugated TCO and albumin conjugated with tetrazine have been used as pretargeting and delivery components, respectively. Both components were labeled with fluorophores without spectral profile conflicts to track with optical imaging. PSMA(+) PC3-PIP and PSMA(–) PC3-Flu cells were used to validate the strategy *in vitro*. The pretargeting component, 5D3(TCO)_8_(AF-488)_2_, labeled the PSMA receptors on the targeted cell surface, and was then followed by a second-component, ALB(DM1)_2.2_(Peg_4_-Tt)_10_(Rhod)_2_ (Figure 2C). The multiple TCO and Tt groups per component, the high density of PSMA receptors per cell surface, and the fast kinetics of the TCO-Tt click reaction are the driving forces for the formation of nanoclusters on the cell surface. An efficient cross-linking of two components on the targeted cell surface leads to enhanced cellular internalization and results in the highest therapeutic effects in PSMA(+) PC3-PIP cells compared to PSMA(–) PC3-Flu cells (Figure 2D). Ideally, the internalization and pharmacokinetics of the pretargeting agent should have similar time scales. After internalization, nanoclusters are hydrolyzed in acidic late endosomes and release cargo drug molecules to the cytoplasm. Eventually, a DM1 anti-tubulin agent blocks the microtubule formation, arresting cell division, and killing targeted cells.

### Pretargeted Theranostics in Colorectal Cancer

Colorectal cancer is the most common cancer type in the world after lung and BrCa (51). The surgical approach of managing colorectal cancer is limited for cases with localized tumors but not feasible in early stage tumors and metastatic tumors. Pretargeting radioimmunotherapy is an optimistic solution to overcome these issues (52). As an initiate, Adumeau et al. (37) has reported a pretargeting multimodal PET/NIRF imaging approach for imaging colorectal cancer. They have used humanized A33 monoclonal antibody (huA33) as the pretargeting platform targeting A33-expressing colorectal cancer. HuA33 was conjugated with TCO and labeled with Dye800 NIR fluorophore and used as the pretargeting component with tetrazine–sarcophagine conjugate labeled with ^64^Cu radionuclide (^64^Cu-Tz-SarAr). This strategy has been successfully evaluated in SM1222 colorectal cancer xenograft tumor mouse models overexpressing A33 cell surface antigen (Figure 2E). Keinanen et al. (38) from the same group have reported the extension of this study from diagnostic [^64^Cu]Cu-SarAr-Tz to therapeutic, using [^177^Lu]Lu-DOTA-PEG_7_-Tz conjugates, as the secondary component. Both [^64^Cu]Cu-SarAr-Tz and [^177^Lu]Lu-DOTA-PEG_7_-Tz have shown high activity in mice models bearing A33-overexpressing subcutaneous tumors (Figure 2F).

The biotin and avidin chemistry has been successfully used for pretargeting imaging of HER2(+) BrCa in preclinical settings (7). Lectin receptors are overexpressed and many cancer types and avidin can bind with lectin as well. Yao et al. (39) have used this phenomenon for pretargeting theranostic approach for image-guided treatment of human colorectal cancer cells, LS180 and HT-29. In their study, avidin without conjugation has been used as the pretargeting component targeting lectin receptors in colorectal cancer cells and tumors in mouse models. Doxorubicin was conjugated with biotic through an acid sensitive hydrazone linker (bDOX). Because of the hydrazone bonding doxorubicin is in prodrug stage. To evaluate the strategy, colorectal cancer LS180 and HT-29 cells were first treated with avidin followed by the administration of bDOX. Avidin is labeled on the lectin receptors of the targeted cell surface. Then biotinylated dDOX will be complexed with avidin followed by rapid internalization by endocytosis. In late endosomes or lysosomes, at low pH acidic condition, hydrazone bond is cleaved releasing free doxorubicin. Compared to doxorubicin alone, pretargeting approach shows higher cellular uptake of the drug. *In vivo* study, LS180 colorectal cancer xenograft mouse models were subsequently administered with avidin and bDOX and significant reduction of tumor growth was observed compared to the mice treated with free doxorubicin (Figure 2G). Hence, this strategy is synthetically convenient and exhibits enhanced therapeutic efficacy with minimal systemic toxicities.

## Conclusions

Theranostics can be a highly important tool in the development of precision, highly efficient, and safe therapy for the personalized treatment of cancer using molecular information from a patient's tumors. The pretargeting approach can leverage the inherent strengths of the theranostic strategy; however, it will require a strategy of orthogonal conjugation in physiological conditions. The combination of pretargeting imaging and therapy provided several advantages over single-component theranostics. This strategy significantly reduces the circulatory time and off-target toxicity of the drug-carrier component and provides objective criteria for optimization of the treatment protocol based on the results of non-invasive imaging. The prerequisites for successful applications of a pretargeting theranostic approach are (a) high expression of the target receptor and the use of a high binding affinity pretargeting component, (b) optimal internalization of the pretargeting component-receptor molecular complex, which is a complex parameters that depends on the tumor microenvironment conditions and should allow an adequate time window for *in situ* reactions with a second delivery component, (c) affinity of the binding of two components on the targeted cell surface, and (d) correct combination of imaging agents/imaging modalities and therapeutics on the therapeutic components.

## Author Contributions

All authors listed have made equal direct and intellectual contribution to the work and preparation of the manuscript and approved the manuscript for publication.

## Conflict of Interest

The authors declare that the research was conducted in the absence of any commercial or financial relationships that could be construed as a potential conflict of interest. The handling editor declared a past co-authorship with the authors.
